# Assessing Measurement Invariance in Neighborhood Socioeconomic Environment Across Levels of Urbanicity

**DOI:** 10.1007/s11524-025-01020-8

**Published:** 2025-10-29

**Authors:** S. S. A. De Silva, Melissa A. Meeker, Tara P. McAlexander, Yasemin Algur, Victoria Ryan, Leslie A. McClure

**Affiliations:** 1 Department of Epidemiology and Biostatistics, Dornsife School of Public Health at Drexel University, Philadelphia, PA 19104 USA; 2 Present Address: Department of Orthopedics and Sports Medicine, Boston Children’s Hospital, Boston, MA 02115 USA; 3 Present Address: Department of Emergency Medicine, Massachusetts General Hospital, Boston, MA 02114 USA; 4 Urban Health Collaborative, Dornsife School of Public Health at Drexel University, Philadelphia, PA 19104 USA; 5 Present Address: College for Public Health and Social Justice, Saint Louis University, St. Louis, MO 63132 USA

**Keywords:** Neighborhood socioeconomic status, Factor analysis, Measurement invariance, Urban health, Rural health

## Abstract

While neighborhood socioeconomic status (NSES) significantly impacts health outcomes, its measurement often assumes that the social and economic mechanisms that drive NSES operate similarly across the urban–rural continuum. This study aimed to develop a census-tract-level NSES measure that accounts for differences across community type and assesses its measurement invariance. Using data from 71,908 census tracts in the contiguous United States, we employed exploratory and confirmatory factor analyses to derive a one-factor NSES construct consisting of five variables: percent below the poverty line, households receiving public assistance, population unemployed, households without cars, and population with less than a high school education. Measurement invariance analysis revealed that while the overall NSES structure is consistent across urban and rural community types, factor loadings varied significantly. The percentage of the population living below poverty was the most reflective indicator across all community types, while other indicators, such as car access and unemployment, exhibited context-specific variability. These findings highlight the importance of incorporating urbanicity into NSES measures in health disparity research and to improve the effectiveness of public health interventions.

## Introduction

Neighborhood socioeconomic status (NSES) is a critical determinant of health outcomes. Research demonstrates that the physical and social dimensions of neighborhoods, such as income, education, employment, and housing, significantly influence the health and wellbeing of individuals [1–3]. Consequently, health disparities are closely associated with an individual’s place of residence, which is often shaped by their social position and ethnicity [3]. However, some evidence suggests that associations between individual level socioeconomic factors and health outcomes may vary by geographic region [4, 5], indicating the importance of considering geography when examining socioeconomic factors. It is conceivable that the impact of socioeconomic indicators is different in rural versus urban areas. For example, not having a car in a rural area could impact health outcomes differently than for those without cars in urban areas [6–8]. Additionally, Xie et al. showed that the association between individual-level and neighborhood socioeconomic factors varies by urbanicity [9], further underscoring the need to better understand the role of geography in NSES. Because policies guiding health interventions are implemented at the neighborhood level, it is important to ensure that measures of NSES that inform policy are sensitive to differences at the community level [10].

The Diabetes Location, Environmental Attributes, and Disparities (LEAD) Network is a Centers for Disease Control and Prevention (CDC)-funded, multisite study designed to determine whether community-level risk factors influence the association between NSES and diabetes among participants in 3 different cohorts [11]. Ultimately, the LEAD Network seeks to provide evidence to inform policies aimed at reducing geographic disparities in diabetes. To better understand the NSES/diabetes relationship, it is necessary to define NSES for use specifically in LEAD analyses, an effort also relevant for other research studies going forward.

The construction of a NSES index typically involves aggregating multiple individual-level measures reflecting different dimensions of socioeconomic status to create a composite measure. For example, Messer et al. [12] developed a census-tract-level NSES index by using principal components analysis (PCA) that retained 8 of 20 census-level variables describing several different NSES domains; and Major et al. [13] similarly used PCA to create an index that retained 10 of 19 potential variables. Xiao et al. [14] adapted those approaches, also using PCA to determine the weightings for 6 neighborhood variable domains. Furthermore, the Area Deprivation Index (ADI), developed by Singh, considered 21 socioeconomic indicators, and using factor analysis, reduced the set to 17 indicators to comprise the index [15]; whereas the Social Deprivation Index (SDI) used factor analysis to retain 7 of 14 measures [16, 17].

However, most composite measures in the literature, including the ADI, the SDI, and those derived by Messer et al. [12], Major et al. [13], and Xiao et al. [14], assume that the social and economic mechanisms that drive neighborhood SES operate similarly across geographic space or the urban–rural continuum. This may not be the case, as suggested by Myint [18], who argues that there may be significant variations in the spatial distributions, patterns, associated functional characteristics, and centrality of socioeconomic factors in urban systems. To explore this argument, we recently compared a non-spatial measure of NSES to a spatial measure that accounted for spatial heterogeneity in the relationship between the socioeconomic variables and the NSES index and found that socioeconomic variables have differential impacts on NSES across counties, and that drivers of NSES may vary across US census regions [19]. These findings are important, as they suggest that a better understanding of NSES in different community types may lead to better public health interventions and policies.

Understanding how NSES operates in different geographic contexts is essential for developing effective policies and interventions to address health disparities. Therefore, the objective of this study is to develop a census tract-level measure of NSES that accounts for variations across the urban–rural continuum and to assess its measurement invariance. Specifically, we aimed to (1) derive a measure of NSES using factor analysis across urban–rural community types, and (2) formally assess whether NSES operates differently across the urban–rural continuum by performing measurement invariance analysis.

## Methods

### Data

We collected census tract-level data on nine neighborhood SES variables for 71,908 census tracts in the contiguous United States from the 2000 US census. We used 2000 Census data to ensure that the exposure data would precede our outcomes in our subsequent analyses. The variables were selected similarly to the development of other indices, such as Messer et al. [12], Xiao et al. [14], the SDI [16], and the ADI [15], and included the following: % of individuals living below the poverty line, % of households with an annual income of less than $30,000, % of households receiving public assistance, median home value, % of unemployed individuals in the population, % of males not in the workforce, % of households without access to a car, % of individuals with less than a high school education, and % of females in management positions. To ensure comparability across variables with different measurement units, we z-score standardized all nine variables prior to analysis.

To assess measurement invariance across the urban–rural continuum, we classified the census tracts using the community type classification derived by the Diabetes LEAD Network, which divides tracts into four categories: higher density urban, lower density urban, suburban/small town, and rural, and better differentiates urban cores from surrounding suburban and non-rural areas. Briefly, the LEAD community type classification was developed using the USDA Rural Urban Commuting Area (RUCA) designations for all US census tracts, which were modified based on tract-level measures of land area from the 2010 US census. Tracts were then classified as urban, suburban/small town, or rural. However, because of the large variation in the sizes of urban tracts, they were further reclassified as high- or low-density urban, again based on the distribution of land area. Thus, the LEAD community type used a combination of population and land area to classify tracts as high density urban, low density urban, suburban/small town, or rural. More details of the community type measure development can be found elsewhere [20].

### Factor Analysis

We used exploratory factor analysis (EFA) to examine the underlying structure of the standardized census variables and identify indicators that could be grouped together to create a measure of NSES. Exploratory factor analysis is applied for scenarios in which the underlying factor structure is unknown [21, 22]. Following the results of the EFA, we used confirmatory factor analysis (CFA) to fit a one-factor model using solely the census variables that displayed high loadings (> 0.60) on the first factor in the EFA and selected which variables to allow residual correlations for based on the bivariate correlations between pairs of variables. To identify the baseline measurement model for the subsequent measurement invariance analysis, we fit variations of the one-factor model, permitting residual correlation among pairs of indicators. We compared the models using the root mean square error (RMSEA) and Bayesian information criterion (BIC) and selected the model with the smallest RMSEA and BIC as the baseline model [23]. Note that some sources indicate that an RMSEA of 0.05 or below is a good fit, values between 0.05 and 0.08 are acceptable [24], values between 0.08 and 1 are marginal, and values greater than 0.1 are poor. However, while the RMSEA is dependent on several factors and there is not universal agreement about a desired magnitude [25], the RMSEA is insensitive to sample size, but sensitive to model complexity [26].

### Assessing Measurement Invariance of NSES by LEAD Community Type

Our study aimed to investigate whether the measurement of the underlying latent NSES variable was consistent across the four LEAD community types. To achieve this, we conducted a measurement invariance analysis. The process involves testing a sequence of nested models that are increasing in restrictiveness, using multigroup confirmatory factor analysis. Briefly, the process begins with assuming configural invariance, which assumes that the same underlying construct is being measured across groups and is required for the other tests [27]. We then test for metric invariance, which further assumes that the indicator loadings are the same across groups [27]. If we fail to reject the presence of metric invariance (*p* > 0.05), we can conclude that the factor loadings differ across groups, and we next test for scalar invariance, which further assumes that the item intercepts are the same across groups [27]. Should we fail to reject scalar invariance (*p* > 0.05), we can conclude that the intercepts differ across groups, and we then test for strict invariance, which further assumes that the factor variances and covariances are the same across groups [27]. If we fail to reject strict invariance (*p* > 0.05), we can conclude that the factor variances and covariances are different across groups as well. Each nested model is compared to the next by conducting a chi-square difference test and by evaluating model fit using the RMSEA. If any of the nested models rejected the null hypothesis of invariance, then the NSES measure is different across the community types: the type of invariance for which the null hypothesis was rejected then informed the degree to which NSES measures differ across community types. We conducted the analysis using the *lavaan* [28] package in R.

## Results

Table 1 summarizes the 9 census variables for each of the community types, as well as across all tracts. Higher density urban areas had the highest percent of people living below the poverty line, households with income less than $30,000, households on public assistance, unemployed individuals, males not in the workforce, households with no car, and a population with less than high school education.

**Table 1:** Summary statistics (mean, standard deviation) of 2000 US census variables by community type

Census variable	All tracts (*N* = 71,908)	Higher density urban (*N* = 17,046)	Lower density urban (*N* = 25,613)	Suburban/small town (*N* = 11,741)	Rural (*N* = 17,508)
% below poverty line	12.9 (11.0)	18.9 (13.7)	10.2 (9.8)	10.2 (9.3)	12.7 (7.8)
% income less than $30 k	35.7 (17.2)	42.0 (18.5)	30.1 (17.0)	31.9 (16.5)	40.3 (12.9)
% public assistance	3.7 (4.2)	6.2 (6.2)	2.8 (3.3)	2.6 (2.8)	3.1 (2.5)
Median home value ($)	132 k (100 k)	155 k (116 k)	147 k (105 k)	130 k (96 k)	91 k (57 k)
% unemployed	6.2 (5.2)	8.8 (6.8)	5.3 (4.6)	5.1 (4.3)	5.6 (3.7)
% males not in workplace	29.3 (11.3)	32.1 (11.6)	26.6 (10.9)	27.7 (11.3)	31.6 (10.4)
% no car	10.5 (12.9)	22.6 (19.5)	7.4 (7.6)	6.0 (5.9)	6.2 (4.4)
% less than high school education	20.3 (13.8)	26.5 (16.8)	16.0 (12.5)	17.1 (11.5)	22.5 (10.4)
% females in management	11.6 (5.9)	11.3 (6.7)	13.2 (6.1)	11.7 (5.4)	9.5 (3.9)

To create a one-factor measure of NSES, we employed an EFA model to group the census indicators. EFA requires specifying the number of factors to extract, which we determined through a parallel analysis that suggested a 3-factor solution was appropriate. Table 2 displays the results of the analysis, presenting the three extracted factors. The first factor had the highest loadings for variables such as percent below the poverty line, percent of households on public assistance, percent of population unemployed, percent of households with no car, and percent of population with less than a high school education.

**Table 2:** Factor loadings from a 3-factor EFA model. The variables that loaded highest on the first factor are indicated with an asterisk

Census variable	Factor 1	Factor 2	Factor 3
% below poverty line	0.80*	0.32	0.32
% income less than $30k	0.54	0.52	0.80
% public assistance	0.80*	0.23	0.09
Median home value ($)	−0.05	−0.70	−0.16
% unemployed	0.73*	0.22	0.13
% males not in workplace	0.47	0.30	0.23
% no car	0.75*	−0.02	0.21
% less than high school education	0.64*	0.50	0.13
% females in management	−0.28	−0.73	−0.10

**Fig. 1:**
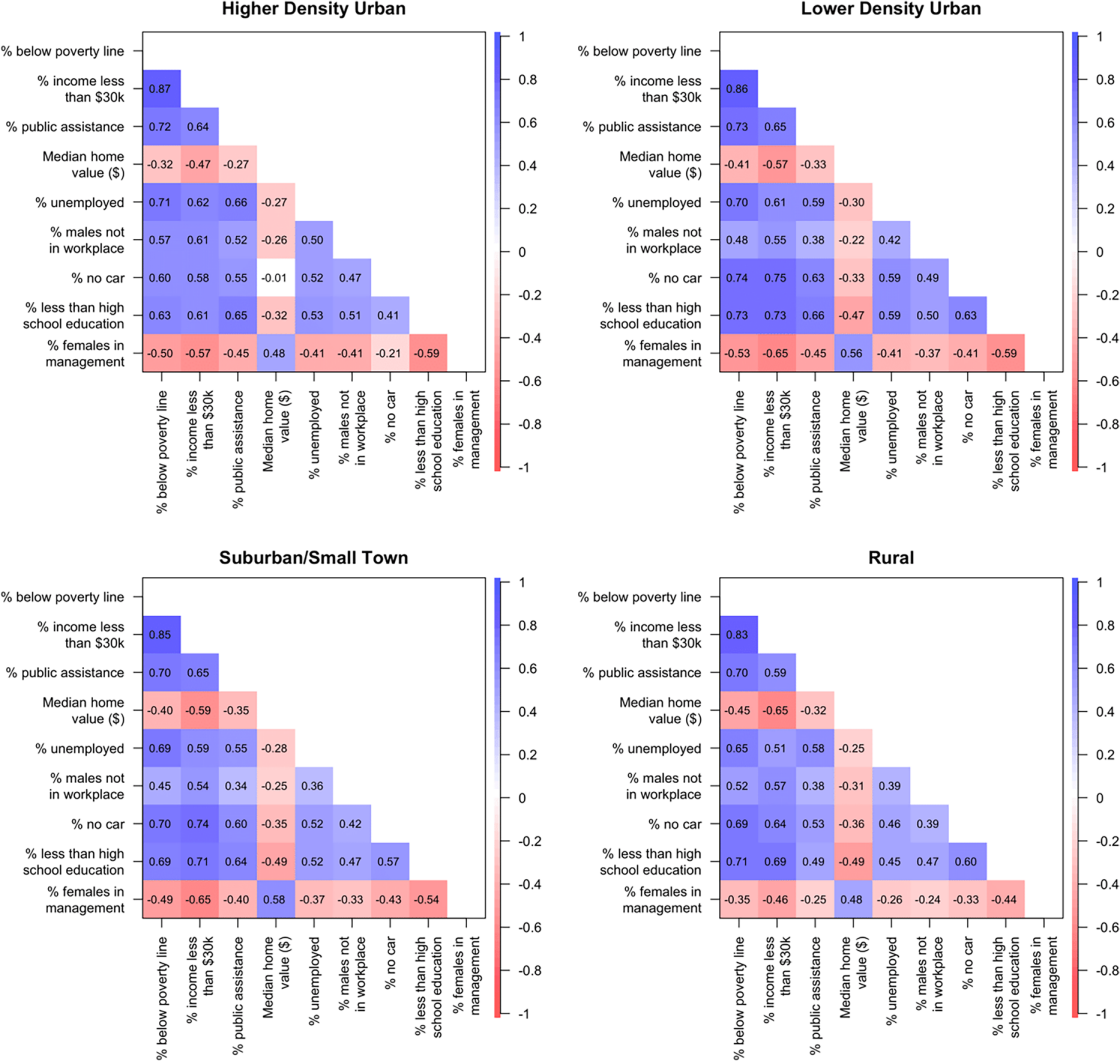
Correlation coefficients between each indicator stratified by community type (higher density urban, lower density urban, suburban/small town, rural)

We then retained the variables with the highest loadings on the first factor from the EFA (asterisks in Table 2) in a CFA to select the baseline model. Since the second and third factors in the EFA were strongly associated with only two variables and one variable, respectively, we prioritized the first factor in the CFA to ensure parsimony and develop a single measure of NSES. Additionally, an evaluation of the model’s RMSEA showed that the one-factor model performed similarly to the 3-factor model, with an RMSEA below 0.8. We fit several one-factor models allowing residual correlations between pairs of the indicators based on variables that showed high raw correlations, defined as correlations greater than 0.7 (Fig. 1). Table 3 shows the RMSEA and BIC from the models. The one-factor model with residual correlation between percent below the poverty line and percent of households on public assistance and between percent unemployed and percent of households on public assistance had the lowest BIC (677,060) and RMSEA (0.06). We therefore selected this model as the baseline model for the measurement invariance analysis.

**Table 3:** Model fit statistics for one factor CFA models representing NSES

One-factor model	BIC	RMSEA
Model with no residual correlation *% below poverty line, % household on public assistance, % unemployed, % no car, % less than HS education*	678,861	0.086
Model with residual correlation between: *% below poverty line and % household on public assistance*	678,168	0.082
Model with residual correlation between: *% below poverty line and % unemployed*	678,380	0.087
Model with residual correlation between: *% below poverty line and % less than HS education*	678,664	0.092
Model with residual correlation between: *% unemployed and % household on public assistance*	677,422	0.065
Model with residual correlation between: *% below poverty line and % household on public assistance* *% unemployed and % household on public assistance*	677,060	0.062

We performed a multigroup confirmatory factor analysis to examine the measurement invariance of the NSES measure across the four community types. We found that the configural model fit significantly better than the metric invariance model (Table 4, *p*-value < 0.001), suggesting that the underlying NSES construct was consistent across all four types of communities (configural invariance was present by assumption); however, the factor loadings of the indicators used for the NSES measure varied significantly among community types (metric invariance was not present since *p* < 0.0001). Because we rejected metric invariance, it was not necessary to test either scalar invariance or strict invariance. Table 5 displays the factor loadings of NSES indicators, which reveal the variation in NSES measurement across community types. Percent below the poverty line had the highest loadings across all four community types, exceeding 0.90. Percent of households with no cars had the lowest loading in higher density urban tracts (0.65), while percent unemployed had the lowest loadings in lower density urban and rural tracts (0.75 and 0.68, respectively). In suburban/small town tracts, the indicator with the lowest loading was percent with less than a high-school degree (0.72). Additionally, the residual covariance loadings showed variation across community types, suggesting that indicator pairs are differentially measuring the NSES construct. Rural communities had the largest residual covariance loadings for both percent below the poverty line and percent on public assistance, as well as percent with less than a high school degree and percent on public assistance.

**Table 4:** Results of multigroup confirmatory factor analysis of NSES across 4 community types

	BIC	RMSEA	*χ*^2^(df)	*p*-value
Configural model	683,811	0.062		
Metric model	683,989	0.133	1603.1 (12)	< 2.2e-16

**Table 5:** Factor loadings of NSES indicators across community type

	Higher density urban	Lower density urban	Suburban/small town	Rural
***Factor loadings***				
% below poverty line	0.92	0.94	0.95	0.96
% public assistance	0.85	0.79	0.78	0.80
% unemployed	0.77	0.75	0.72	0.68
% households with no car	0.65	0.80	0.75	0.71
% less than HS degree	0.68	0.78	0.73	0.75
***Residual covariance loadings***				
% below poverty line and % public assistance	− 0.28	− 0.07	− 0.02	− 0.42
% less than high-school degree and % public assistance	0.19	0.11	0.04	− 0.28

## Discussion

In this study, we used factor analysis to develop a composite measure of NSES and explored its potential variations across urban and rural community types through measurement invariance analysis. Our findings revealed a one-factor NSES construct, encompassing the percent living below the poverty line, percent of households receiving public assistance, percent of the population unemployed, percent of households without cars, and percent of the population with less than a high school education. Notably, the measurement invariance analysis indicated that while the factor structure is the same across community types, there are significant differences in factor loadings in the NSES measure across community types, suggesting that the association between the NSES measure and underlying socioeconomic indicators might vary depending on the urban–rural context. In our analysis, the percent of individuals living below the poverty line was most highly reflective of NSES across all communities. However, the percent of households without a car was highly reflective of NSES in lower-density urban communities, but less so in higher-density urban areas. In higher-density urban communities, the percent of unemployed individuals had a stronger influence on the NSES construct, whereas in rural communities, it was less indicative of socioeconomic status.

Several authors have examined NSES in the context of census tracts, and there is considerable overlap in the variables that inform the NSES measure across studies. Some commonly used measures that are often cited include the Social Vulnerability Index (SVI) [29], the Social Deprivation Index (SDI) [16, 17], and the Area Deprivation Index (ADI) [15, 30]. Like our measure of NSES, each of these was developed using a variety of demographic factors, many of which overlap. For example, the SVI uses 16 indicators from the census, including race and ethnicity, which we did not consider in our measure; additionally, the methodology differs in that it includes all 16 indicators and percentile-ranks them. Furthermore, the SVI has a larger focus on household characteristics than we did, which is necessary to address the needs of communities during disasters. Similar to our measure, the SDI includes 7 census variables, again with some overlap. The methodology used to develop the SDI is similar to ours as well; it uses a factor analysis to reduce the set of indicators from 16 to 7 by only including indicators with a loading of 0.60 or greater. However, the SDI is based on data from the 2005–2009 American Community Survey and thus did not provide an exposure that preceded the initiation of our cohorts. The ADI, which provides a relative measure comparing neighborhoods to each other and uses variables that share some similarities to ours, is only available at the census block group and has not been validated on other scales; thus, it did not suit the needs of the LEAD study.

Other authors who have considered NSES measures include Messer et al. [12], who examined a set of 20 potential census tract variables to include in the NSES index. After employing a PCA, they reduced their set of indicators to eight: percent of males in management and professional occupations, percent of crowded housing, percent of households in poverty, percent of female-headed households with dependents, percent of households on public assistance, and households earning < $30,000 per year estimating poverty, percent earning less than a high school education, and the percent unemployed. While these variables are similar to the indicators we examined, they do not take into consideration community type in their index. In addition, Major et al. [13] also examined 19 indicators, and using PCA, reduced them to a set of 10: percent with less than high school, percent of non-Hispanic blacks, percent unemployed, percent of females in management, percent of males in management, percent of households with income (1999) below poverty, percent female head of household, percent of households with public assistance income, percent of households with income < $30,000, and percent of households with no vehicle, again presenting similar indicators to those examined herein. While this research team did consider the consistency of their PCA results across states, they ultimately presented an index derived by pooling across states, and thus did not consider the impact of rurality on their index.

NSES is an important factor to consider when examining modifiable risk factors for disease, and several study groups have used it in this fashion [12, 13, 31, 32], including our own Diabetes LEAD Network Team [33]. However, none of these studies have considered the impact of the community type on the development of their measure of NSES. Our results indicate the importance of doing so. By considering community type when developing measures of NSES, more targeted interventions and policies can be developed and implemented that take into consideration the unique characteristics of the communities. In fact, our results suggest that not considering community type in the NSES measures could lead to misclassification of exposure that could be differential by community type or other important geographic contexts. In the context of the LEAD study, which was implemented to try to better understand factors that contribute to geographic disparities in new diabetes onset, with a goal of informing policy, recommended policies and policy changes may not impact the communities in the ways intended. In turn, this could lead to misuse of resources.

To our knowledge, we are the first to identify the lack of measurement invariance in NSES; however, our work has limitations. First, our analysis was limited to a narrow set of census variables, which may not capture the full range of socioeconomic processes at play. While we selected our variables based on examples from the literature, it is possible that we missed critical neighborhood measures. To gain a more comprehensive understanding of these processes, future studies should explore additional neighborhood characteristics such as environmental factors, safety, and social capital, to gain a more comprehensive understanding of NSES, similar to the work of Mujahid et al. [34]. Additionally, it is worth noting that census tracts may not fully represent an individual's neighborhood. For example, administrative boundaries may not align with people’s conceptions of neighborhood boundaries, and individuals often interact with and are affected by areas beyond their immediate census tract. Lastly, the extent to which the NSES measure varies across community type depends on how community type is defined, and results may vary as the definition varies.

Our study also has several strengths. To our knowledge, it is the first study to assess invariance in a measure of NSES across the urban–rural context, highlighting the importance of considering community type in understanding socioeconomic mechanisms. The findings suggest that a stratified approach to constructing NSES may provide a more nuanced understanding of variations in NSES across different types of communities, which could aid policymakers and public health practitioners in tailoring interventions and policies to address disparities, as well as help minimize misclassification of NSES in different geographic contexts.

In summary, our study highlights the variability of NSES measurement across community types, emphasizing the need for tailored approaches in public health interventions. Without considering community type when assessing health outcomes as a function of NSES, it could lead to misclassification of associations and perhaps less-than-optimal implementation of policies aimed at improving health outcomes. Future research should explore additional variables and contexts to further understand these dynamics.

## Declarations

### Declaration of AI and AI-Assisted Technologies in the Writing Process

During the preparation of this work, the authors did not use any AI or AI-assisted technologies.
